# Supplementary figures and images for: Comparative proteomics reveals Cryptosporidium parvum manipulation of the host cell molecular expression and immune response
Source: PLoS Negl Trop Dis. 2021 Nov 24;15(11):e0009949. doi: 10.1371/journal.pntd.0009949 (PMC8612570; doi:10.1371/journal.pntd.0009949)

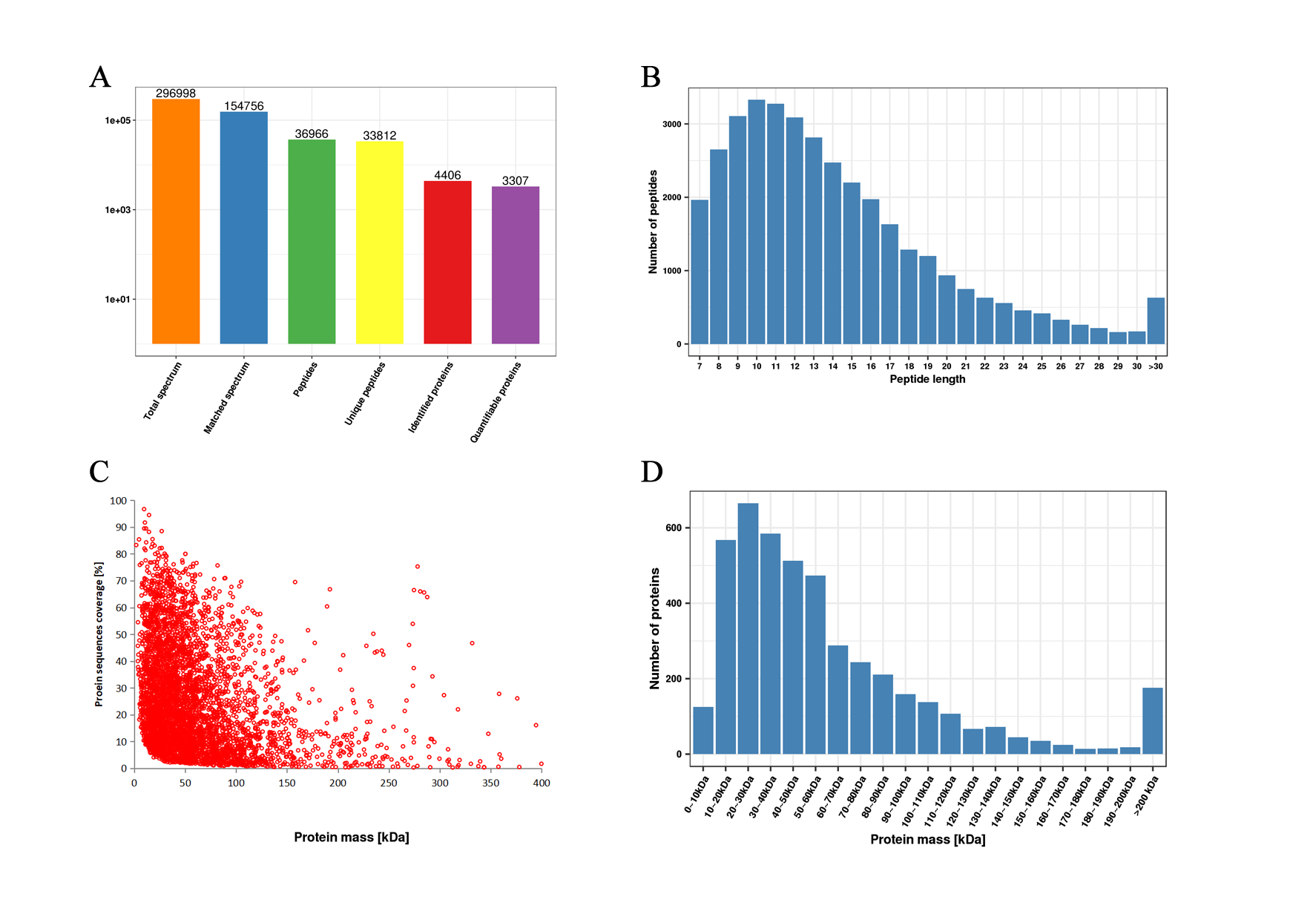

Supplement: S1 Fig — (A) Basic statistics of the mass spectrometry data. (B) Length distribution of identified host peptides. (C) Mass and sequences coverage ratio of host proteins. (D) Mass distribution of identified proteins in host cells. (TIF) [file pntd.0009949.s002.tif]

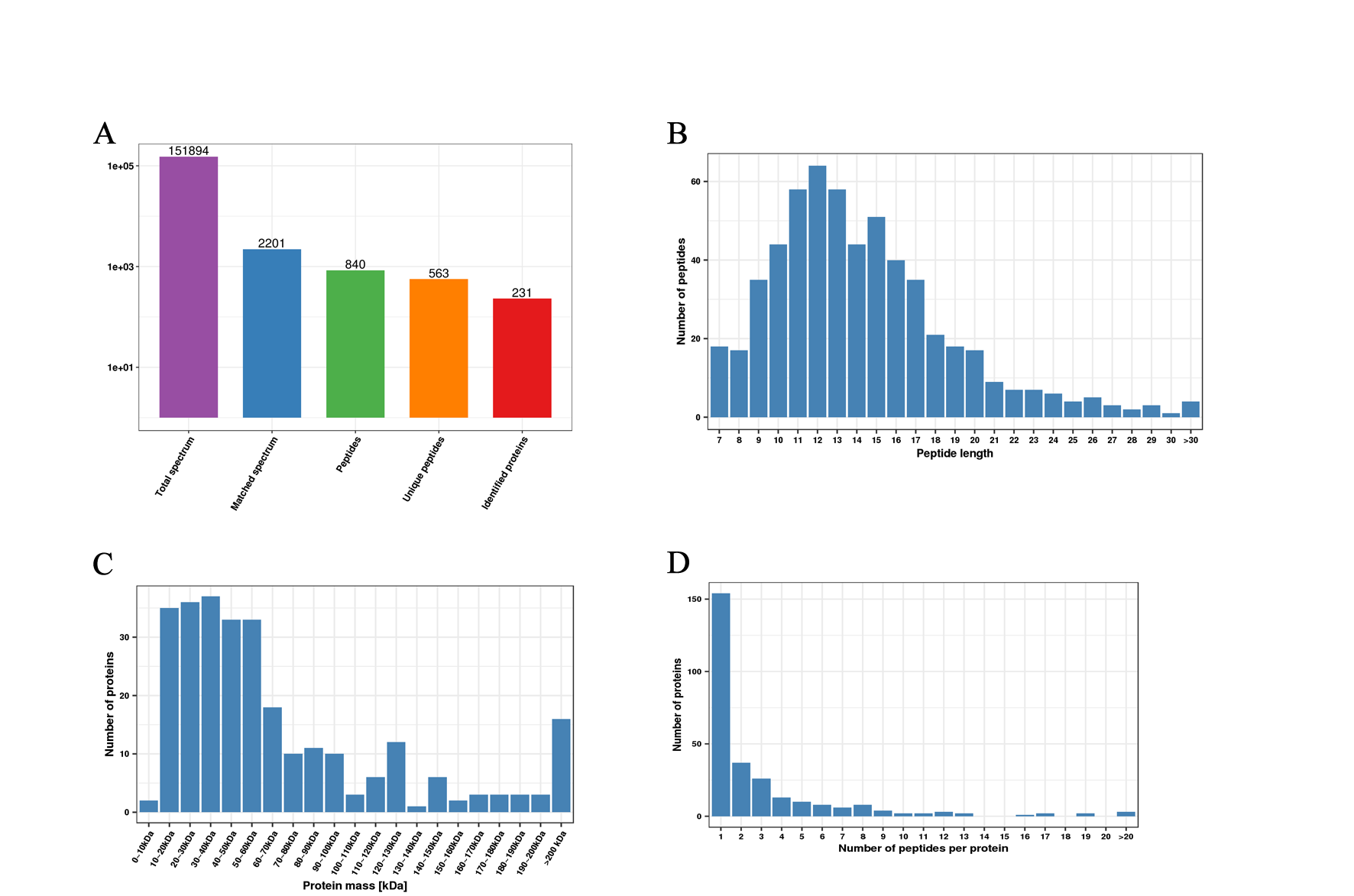

Supplement: S2 Fig — (A) Basic statistics of the mass spectrometry data of identified C. parvum proteins. (B) Length distribution of identified C. parvum derived-peptides from host cells. (C) Mass distribution of identified C. parvum proteins from host cells. (D) Distribution of the number of peptides per protein. (TIF) [file pntd.0009949.s003.tif]

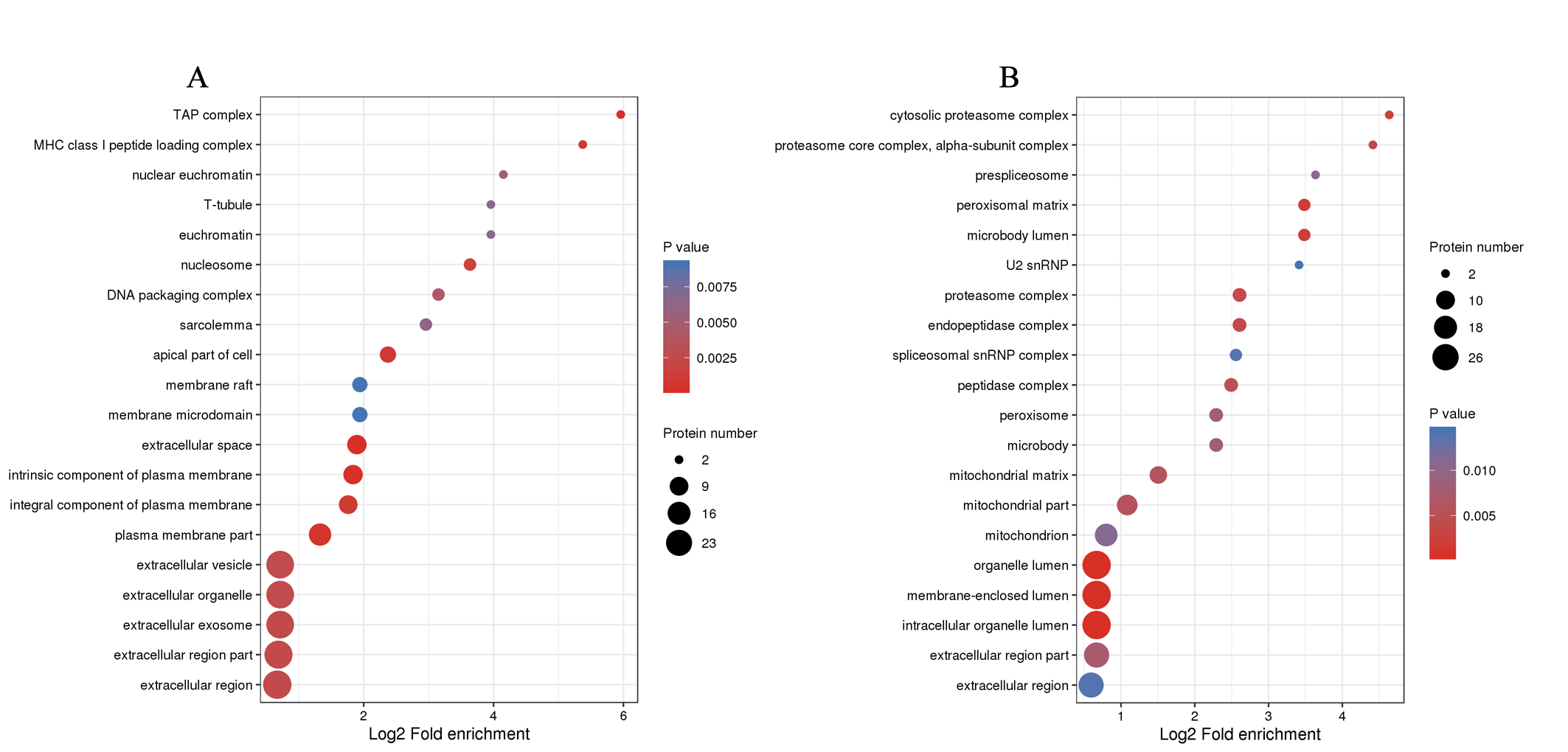

Supplement: S3 Fig — GO enrichment analysis of upregulated (A) and downregulated proteins (B) in the Cellular Component category. The size of the circle area represents the number of DEPs, and the color represents the P value of the enrichment significance of the DEPs under the GO classification. The redder and more distributed to the right the circles, the more important their classification. (TIF) [file pntd.0009949.s004.tif]

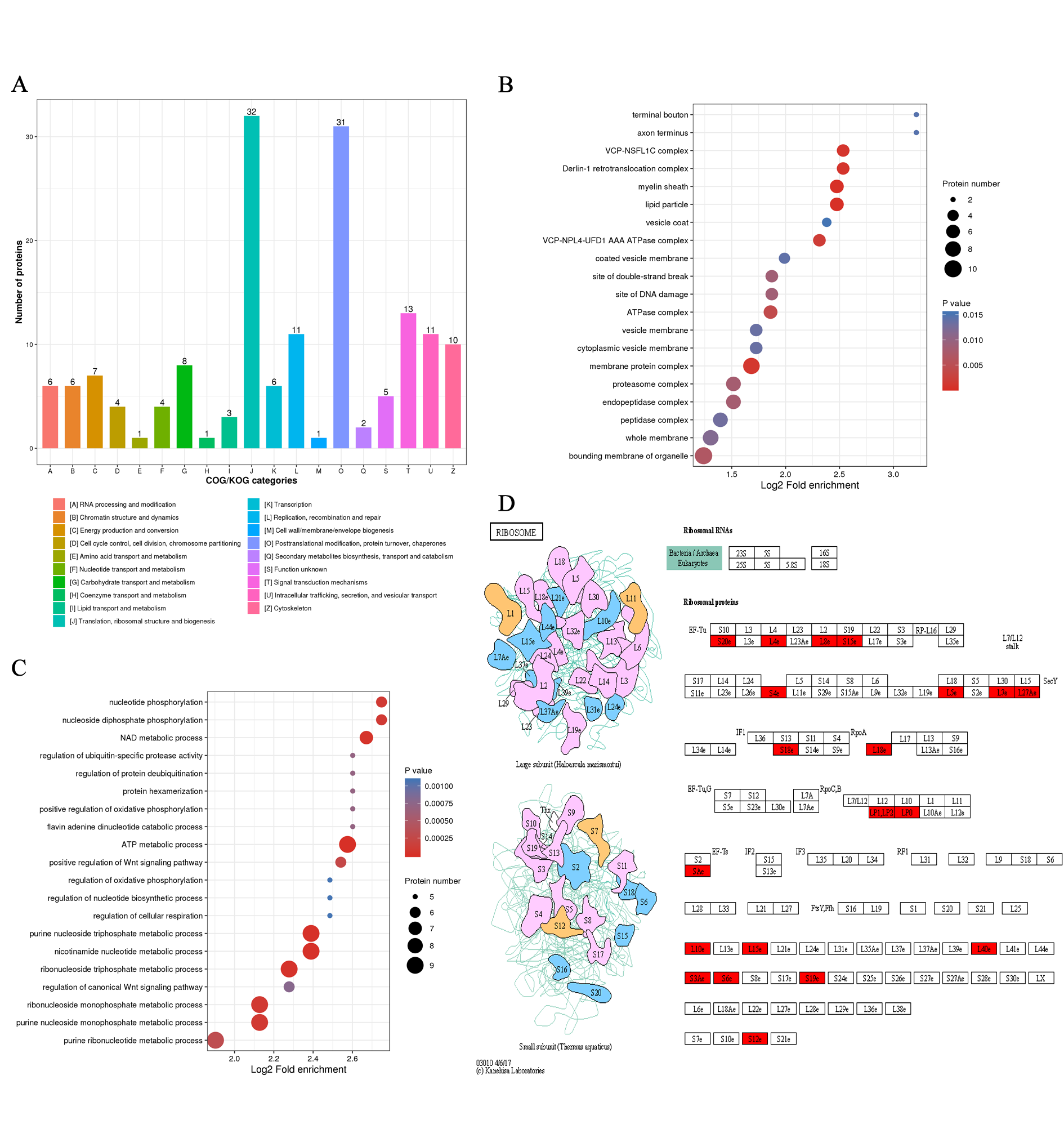

Supplement: S4 Fig — (A) COG/KOG analysis of identified C. parvum proteins. (B) GO enrichment analysis of parasite-derived proteins. The size of the circle represents the number of DEPs in that functional class or pathway, and the color represents the significance of the enrichment (P value). (C) GO enrichment analysis of identified C. parvum proteins in the Biological Process category. (D) C. parvum-derived proteins that were enriched significantly in the ribosome pathway from the KEGG pathway analysis. Protein names in red are parasite-derived proteins identified from infected HCT-8 cells. (TIF) [file pntd.0009949.s005.tif]

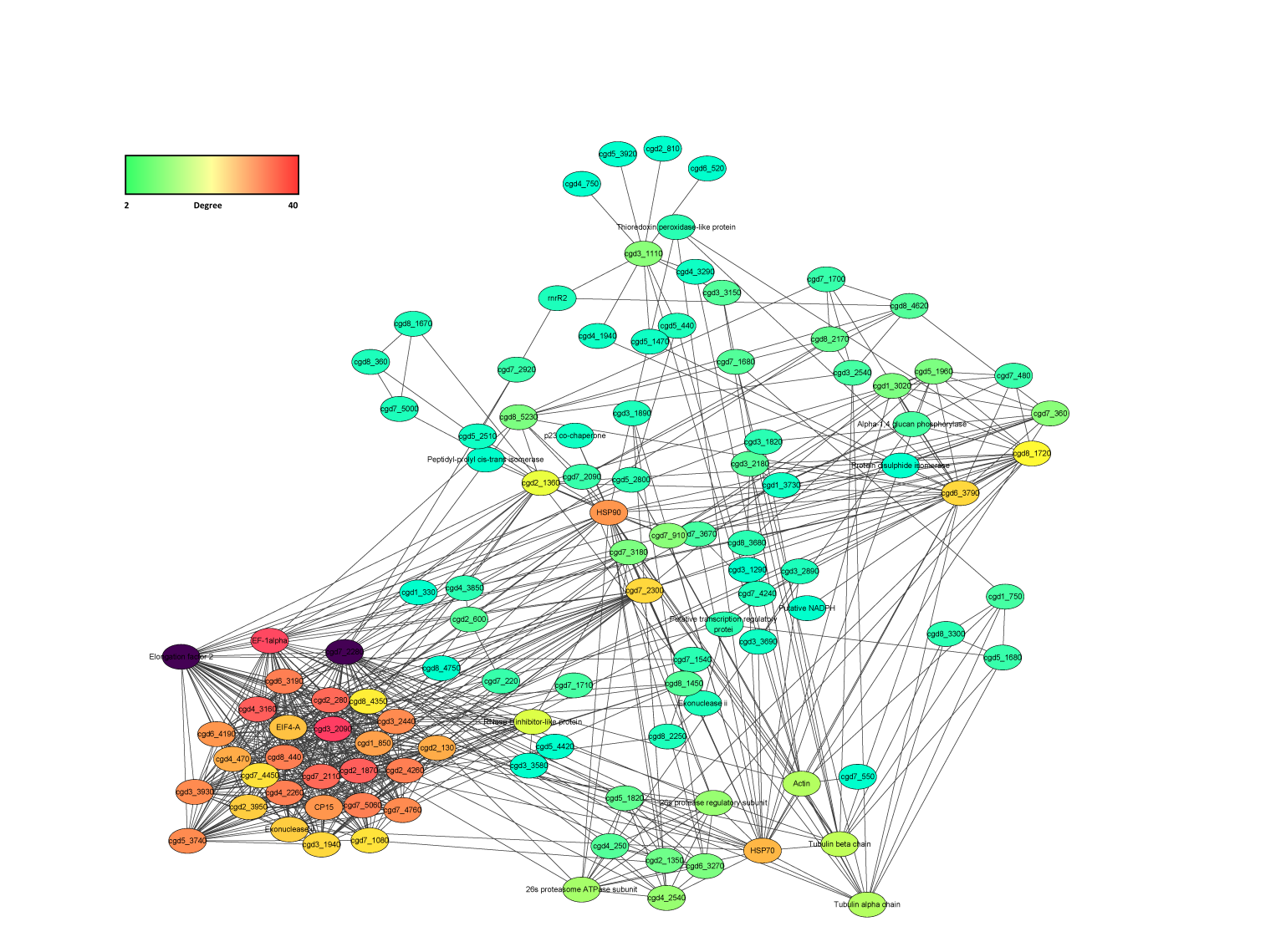

Supplement: S5 Fig — The degree represents the strength of the protein interaction. (TIF) [file pntd.0009949.s006.tif]
