# Supplementary material for: Comparative proteomics reveals Cryptosporidium parvum manipulation of the host cell molecular expression and immune response
Source: PLoS Negl Trop Dis. 2021 Nov 24;15(11):e0009949. doi: 10.1371/journal.pntd.0009949 (PMC8612570; doi:10.1371/journal.pntd.0009949)
Supplement: Attachment — Submitted filename: Responses.docx [file pntd.0009949.s007.docx]

**Dear Editor and Reviewers,**

Thank you for the helpful feedback, and the opportunity to revise and improve our manuscript. We have carefully revised our manuscript, including thorough English language editing, and have revised the results section. Below is a point-by-point response to all of the reviewers’ comments (R1 and C1 are indicated in the revised manuscript with a highlighted version with changes and the clean version, respectively). We would like to thank you and the referees again for the valuable comments and suggestions.

Reviewer's Responses to Questions

Key Review Criteria Required for Acceptance?

As you describe the new analyses required for acceptance, please consider the following:

**Methods**

-Are the objectives of the study clearly articulated with a clear testable hypothesis stated?

-Is the study design appropriate to address the stated objectives?

-Is the population clearly described and appropriate for the hypothesis being tested?

-Is the sample size sufficient to ensure adequate power to address the hypothesis being tested?

-Were correct statistical analysis used to support conclusions?

-Are there concerns about ethical or regulatory requirements being met?

Reviewer #1: THIS IS A WELL CRAFTED STUDY, AND UNQIUE LOOK AT C. parvum's AFFECT ON THE HOST. THEY UTILIZE HCT-8 cells AS A MODEL FOR ISC, AND THE STUDY LOOKS A DEP's AND HOW THIS AFFECTS THE HOST CELL. IT IS AN EXCELLENT STUDY WITH EXCELLENT CONTROLS TO PROVE THEIR DATA. THE DATE WAS ANALYZED  BY SEVERAL MEANS AND I FOUND THEIR DATA AND ANALYSIS QUITE CONPELLING.

**Answer:** Thank you for your appreciation of our methods.

Reviewer #2: Methods fully explained

**Answer:** Thank you for your appreciation of our methods.

**Results**

-Does the analysis presented match the analysis plan?

-Are the results clearly and completely presented?

-Are the figures (Tables, Images) of sufficient quality for clarity?

Reviewer #2: Yes the results match the plan. But the mass of results ultimately leads to a manuscript that reads like a table rather than a concise and focused manuscript.

**Answer：**Thank you for this insightful comment. We have revised our manuscript according to your helpful suggestion. Thank you for highlighting this issue and helping us to improve our manuscript.

**Conclusions**

-Are the conclusions supported by the data presented?

-Are the limitations of analysis clearly described?

-Do the authors discuss how these data can be helpful to advance our understanding of the topic under study?

-Is public health relevance addressed?

Reviewer #2: There are lots of results, but no real conclusion:  from the abstract "This systematic analysis of the proteomic of C parvum-infected HCT-8 cell identified a wide range of functional proteins that participate in host anti-parasite immunity or act as potential targets during infection, providing a new insight into the molecular mechanism of C parvum infection. "

**Answer：**Thank you for highlighting this issue. In this study we aimed to fully determine the detailed interaction between the host and *C. parvum*, and identified several DEPs and signaling pathways that might play important roles during *C. parvum* infection. Based on the high throughput bioinformatic analysis in this study, we provide some new understanding and ideas for *C. parvum* infection and candidate therapies. We have revised our discussion and clarified the conclusion. Besides, we are now exploring the specific roles of several molecules identified from proteomic data for future publication.

**Editorial and Data Presentation Modifications?**

Use this section for editorial suggestions as well as relatively minor modifications of existing data that would enhance clarity. If the only modifications needed are minor and/or editorial, you may wish to recommend “Minor Revision” or “Accept”.

Reviewer #1: (No Response)

Reviewer #2: Fine overll. There are singular words that should be plural and plural words hat should be singular.

**Answer：**Thank you for your helpful suggestion for the improvement of our manuscript. We have carefully revised our manuscript, including thorough English language editing.

**Summary and General Comments**

Use this section to provide overall comments, discuss strengths/weaknesses of the study, novelty, significance, general execution and scholarship. You may also include additional comments for the author, including concerns about dual publication, research ethics, or publication ethics. If requesting major revision, please articulate the new experiments that are needed.

Reviewer #2: The strength of the work is the work that was done.  The weakness is that it reads like a list in the methods and in the discussion.  However, greet data that should be shared.

**Answer：**Thank you for highlighting this issue. We have revised our manuscript carefully according to your excellent suggestion.

We would like to thank you again.

Sincerely yours,

Dr. Jian-Ping Cao, M.D., Ph.D.

Professor of Immunology and Parasitology

National Institute of Parasitic Diseases, Chinese Center for Disease Control and Prevention (Chinese Center for Tropical Diseases Research)

207 Rui Jin 2nd Road, Shanghai 200025, P.R. China

Email: caojp@yahoo.com, caojp@chinacdc.cn
