# Supplementary material for: Comparative proteomics reveals Cryptosporidium parvum manipulation of the host cell molecular expression and immune response
Source: PLoS Negl Trop Dis. 2021 Nov 24;15(11):e0009949. doi: 10.1371/journal.pntd.0009949 (PMC8612570; doi:10.1371/journal.pntd.0009949)
Supplement: Attachment — Submitted filename: Responses.docx [file pntd.0009949.s008.docx]

**Dear Editor and Reviewers,**

Thank you for the helpful feedback, and the opportunity to revise and improve our manuscript. We have carefully revised our manuscript, including thorough English language editing, and have revised the results section. Below is a point-by-point response to all of the reviewers’ comments (R1 and C1 are indicated in the revised manuscript with a highlighted version with changes and the clean version, respectively). We would like to thank you and the referees again for the valuable comments and suggestions.

1. The results match the plan but the mass of results ultimately lead to a manuscript that reads like a table rather than a concise and focused manuscript.

**Answer:** Thank you for your helpful suggestion for the improvement of our manuscript. In this study we aimed to fully determine the detailed interaction between the host and *C. parvum*, and identified several DEPs and signaling pathways that might play important roles during *C. parvum* infection. Based on the high throughput bioinformatic analysis used in this study, we provide some new insights and ideas for *C. parvum* infection and candidate therapies. We have carefully revised our manuscript according to your suggestion and clarified the conclusion.

2- There are singular words that should be plural and plural words that should be singular.

**Answer:** Thank you for your helpful suggestion. We have carefully revised our manuscript, including thorough English language editing.

3- The weakness is that the manuscript reads like a list in the methods and in the discussion and should be revised.

**Answer:** Thank you for this insightful comment. We have revised the methods part and clarified the discussion in our manuscript according to your helpful suggestion. Thank you for highlighting this issue and helping us to improve our manuscript.

We would like to thank you again.

Sincerely yours,

Dr. Jianping Cao, M.D., Ph.D.

Professor of Immunology and Parasitology

National Institute of Parasitic Diseases, Chinese Center for Disease Control and Prevention (Chinese Center for Tropical Diseases Research)

No. 207 Rui Jin 2^nd^ Road, Shanghai 200025, P.R. China

Email: caojp@yahoo.com, caojp@chinacdc.cn
